# How to Effectively Display Sponsorship Information: The Influences of External Time Cues and Information Type on Individuals’ Evaluations

**DOI:** 10.3389/fpsyg.2022.786676

**Published:** 2022-02-09

**Authors:** Yuan Zhang

**Affiliations:** Business School, Huaqiao University, Quanzhou, China

**Keywords:** temporal framing, sponsorship-linked marketing, sponsor information type, global processing style, local processing style

## Abstract

Time, an important, yet scarce resource in daily living, affects cognition, decision-making, and behavior in various ways. For instance, in marketing practice, time-bound strategies are often employed to influence consumer behavior. Thus, understanding and mastering a target market from a temporal perspective can contribute to the ease with which marketers and businesses formulate marketing strategies. Accordingly, this research conducts three studies to explore the influence of temporal framing as an external time cue on the evaluation of sponsorship-linked marketing campaigns. The studies show that future-framed participants adopted a global processing style. In this context, providing information about the sponsoring brand and sponsored event induced a more positive evaluation of future campaigns. However, in a past-frame context, participants were less likely to adopt a global processing style. Here, providing brand sponsor information alone increased the likelihood of a positive evaluation of past campaigns. Ultimately, the findings provide a theoretical basis for decision-making utilizing the influence of activities and events to enhance brand image.

## Introduction

As a critical emerging strategy in marketing communications, sponsorship-linked marketing helps corporations connect sponsored activities, media channels, and target audiences to build brand equity and maximize sponsorship investment effectiveness (Woisetschläger et al., 2017; Cornwell and Kwon, 2020; Alonso Dos Santos et al., 2021). Considered the largest emerging market, China shows increasing potential in such sponsorships. According to the report “Changes in sponsorship value – global sports marketing trend in 2021” from AC Nielsen, Chinese brands will account for one-third of the growth of the global sponsorship market in the next 10 years. Since the 2008 Beijing Olympic Games, Chinese enterprises are increasingly utilizing sport sponsorship as a platform to support their brand growth. From 2015 to 2019, the sponsorship expenditure of Chinese brands increased at a compound annual growth rate of approximately 8.9%, far exceeding its competitors in Europe and the United States. Hisense, a Chinese home electronics brand, significantly increased its sponsorship expenditure for the 2018 World Cup and a FIFA sponsor, Vivo, increased it expenditure for the 2020 and 2024 European Cup. In the top twelve sponsors of the 2021 European Cup, China held four seats, including Hisense, Alipay, Vivo, and TikTok, thereby becoming the country with the largest number of top sponsors of this Cup. According to Deloitte’s report in 2018, even in China’s domestic market, the scale of sponsorship marketing will reach 4.24 billion yuan in 2022, of which the scale of football sponsorship will reach 2.27 billion yuan, accounting for 54% of the total. How to fully utilize sponsored events involving substantial capital investment, is a major concern for marketers who want to ensure the effectiveness of sponsorship campaigns.

Moreover, as sponsorship is a Marketing Science Institute MSI (2020–2022) research priority, it has attracted considerable scholarly attention. Currently, research in the sponsorship-linked marketing domain mainly addresses the firm and individual levels. At the firm level, researchers focus on how corporate performance is affected, such as the relationship between sponsorship-linked marketing and return on investment (Clark et al., 2002; Jensen and Cobbs, 2014; Jensen, 2017), and announcements of sponsored events and abnormal returns (Mazodier and Merunka, 2012; Mazodier and Rezaee, 2013). At the individual level, studies address the relationship between sponsorship-linked marketing and brand equity (Messner and Reinhard, 2012; Cornwell, 2019; Alonso Dos Santos et al., 2021), image transfer between the sponsoring brand and the sponsored event in sponsorship (Alonso-Dos-Santos et al., 2016; Alonso Dos Santos et al., 2020), and the impact of information types on consumer attitudes toward sponsorship (Baek and Reid, 2013; Woisetschläger et al., 2017).

Cornwell and Kwon (2020) noted that how consumers process sponsorship-linked marketing information impacts brand recognition and the effectiveness of the sponsorship campaign, further affecting corporate performance. As individuals adopt different information processing strategies when faced with events at different periods (past, present, and future), their preferences for sponsorship information are also likely to vary. In fact, it is well known that the effectiveness of sponsorship-linked marketing develops over time, and it is so successful at establishing links between the sponsoring brand and sponsored event that it has carryover effects in which people may recall the sponsorship relationship for some time even after the relationship has ended (Edeling et al., 2017; Cornwell, 2019; Alonso Dos Santos et al., 2021). Hence, how to enhance the effectiveness of different types of information at various stages of a sponsorship-linked marketing campaign is particularly important. Although some studies investigate the impact of timing on sponsorship-linked marketing, such as the timing of sponsorship news releases (Clark et al., 2002), most focus on reactions to in-stock markets (Cornwell et al., 2001, 2005a,b; Clark et al., 2002). However, individuals’ reaction to temporal information remains inadequately explored. Further, there are no in-depth studies that investigate people’s preference for information processing strategies when sponsorship-linked marketing occurs at different time points. Hence, this research examines the effect of temporal framing on processing style in the context of the effectiveness of sponsorship information types.

Individuals receive temporal stimuli every day, including advertisements of new products before or after market launches (Grant and Tybout, 2008; Zhao et al., 2014). Temporal framing impacts how individuals pay attention to phenomena, which, in turn, affects judgment and cognition (Weick, 1979; Gilbert and Malone, 1995). How does temporal framing affect individuals’ interpretation of events? Previous theories show a long-standing interest in various perspectives of time; however, the majority have focused on signal temporal framing, such as the future or the past (Lee et al., 2017; Xu and Jin, 2021). What cannot be ignored is that individuals may prefer different types of information (the actor and the situation), when temporal frames vary. People may overestimate the inevitability of outcomes after events have occurred (Fischhoff, 1975) and reveal how actors and observers explain past events (Jones and Harris, 1967). Past studies also show that people tend to focus more on specific subject information when events have occurred in the past (Gilbert and Malone, 1995), while the event’s actor and context are more holistically emphasized when set to occur in the future (Grant and Tybout, 2008). These prior studies reveal systematic differences attributable to temporal framing, which provides a starting point for the hypothesis that individuals may focus more on situational contingencies in the future as compared to the past.

It is noteworthy that prior research provides much insight into temporal *distance’s* effect on feeling, thinking, and behaving. Research on construal level theory (CLT) documents how both the mental representation and goals associated with an event vary as a function of temporal distance (Liberman and Trope, 1998; Liberman et al., 2002; Trope and Liberman, 2003). CLT posits that people represent temporally distant events at a higher, more abstract construal level, whereas they represent temporal proximate events at a lower, more concrete construal level, both in the future and past (Trope and Liberman, 2003). However, they focus more on the psychological distance (near or far), pay less attention to the changing rate of the construal level, and even provide limited insight into temporal *framing*’s effect, which may have the same time interval. From the perspective of information processing strategy, individuals may present different degrees of adopting a processing style with varying temporal framings. Pursuant to the idea that there exists both retrospection and prospection in temporal thinking — and this paper suggests that there are — existing theories of temporal distance may be descriptively incomplete and in need of improvement and replenishment (Van Boven et al., 2009).

Accordingly, the current research extends previous studies and introduces temporal framing as an external time cue (past and future) to explore how information types of sponsorship-linked marketing campaigns affect individuals’ evaluations. Further, prior studies show that temporal framing has a relationship with processing styles (Weick, 1979; Grant and Tybout, 2008; Van Boven et al., 2009). Therefore, temporal framing is assumed to affect the ability to adopt a global processing style, impacting the effectiveness of a sponsorship campaign when presented with different types of information. Principally, this research employs the global-local processing style theory to unveil the mechanisms under the effects of temporal framing and information types on sponsorship campaign evaluations. Prior studies have found that global-local processing style substantially impacts individual behavior, including the preference for product assortments (Goodman and Malkoc, 2012), motivational orientations of approach and avoidance (Nussinson et al., 2011), atypical product evaluations (Förster and Denzler, 2012), the pursuit of goals (Etkin and Ratner, 2013), and time-bound discounts (Kim et al., 2013). However, questions, such as how global-local processing style affects the preference for different types of sponsorship information and whether such styles affect sponsorship-linked marketing evaluations, remain unanswered. The relationship between temporal framing and processing style also remains untested. In summary, this research theoretically and empirically explores the temporal interaction effect and the underlying mechanism, which provided novel evidence of the effectiveness of the sponsorship-linked marketing (Alonso Dos Santos et al., 2020, 2021).

First, according to the theory of the psychology of time, the current research explored how individuals focus on information regarding past and future events, hypothesizing that individuals are likely to focus on the actor of a past event, while focusing more on both the actor and situation of future events. Next, the research investigated the effect of temporal framing and information types on individuals’ evaluation of sponsorship-linked marketing campaigns. A review of past studies shows that providing information about the sponsoring brand is conducive to improving individuals’ evaluation of past campaigns; meanwhile, providing information, both on the sponsoring brand and sponsored event, helps improve the evaluation of future campaigns. Then, the research introduced the global-local processing style theory to discover the underlying mechanism, noting that, relative to past campaigns, future campaigns are more likely to prompt the adoption of a global processing style. As a result, information regarding the sponsoring brand and sponsored event is required for evaluation when the campaign is framed in the future.

Empirically, the current research conducted three studies to test the effect of temporal framing and information types on individuals’ evaluation of sponsorship-linked marketing campaigns. Textual information was used to manipulate temporal framing (past and future) and rule out the alternative explanations of processing fluency and perceived uncertainty. Moreover, a figure identification task, where processing style was manipulated, was employed to test the underlying mechanism of global processing style.

## Research Hypotheses and Theoretical Framework

### Interaction Effect of Temporal Framing and Information Type on Consumers’ Evaluation

Research on the psychology of time has shown that time framing affects how individuals pay attention to events. Weick (1979) investigated how participants with different temporal orientations describe sports games and found that, relative to past-oriented participants, those with a future orientation described the game more diversely and included player descriptions and a broader view of the game. In another experiment, participants were required to describe the scene of a car accident. He found that future-oriented participants described the accident within a more general context, while past-oriented participants focused on specific details, such as the vehicle and its occupants (Weick, 1979). Bavelas (1973) revealed similar results; when asked to make a travel itinerary for an unfamiliar professor, future-oriented students considered more travel destinations than past-oriented students did. These studies show that when individuals think about a future event, they will first consider what is happening because future events are flexible, and then react accordingly, based on their thinking (Weick, 1979). Additionally, people preferred to make more choices in a much broader set when they thought of a future event (Zhang and Guo, 2019). In the word-of-mouth domain, people are more likely to share information if the event is happening in the future (vs. the past) and topics reflect well on the sharer (Weingarten and Berger, 2017). Hence, future events are placed in a broader and more comprehensive context (Weick, 1979; Grant and Tybout, 2008; Zhao et al., 2014). In summary, in the context of sponsorship-linked marketing, this research inferred that, relative to the sponsorship-linked marketing campaign that has occurred in the past, people will evaluate the sponsorship-linked marketing campaign in the future based on broader considerations and more comprehensive data, which may include information about both the sponsoring brand and sponsored event.

Grant and Tybout (2008) highlighted that event descriptions can be characterized in terms of the actor (e.g., a sponsoring brand, who leads the sponsorship-linked marketing event) and situation in which the event occurs (e.g., the market environment and background of sponsored events, which present the place or context that the sponsoring brand takes actions in). According to the attribution bias theory, when considering past events, individuals tend to focus narrowly on the actor (Jones and Harris, 1967; Gilbert et al., 1988; Gilbert and Malone, 1995; Ross et al., 2005). For instance, if consumers experienced a failed dining service, they usually attributed the failure to the waiter, rather than the restaurant environment (Folkes, 1984). Accordingly, the hindsight bias theory affirms that individuals tend to focus on the actor when considering past events. Hawkins and Hastie (1990) discovered that, when evaluating the outcome of a past battle, descriptions of the victorious army (the actor) determined the evaluation; however, a description of the opponent and the terrain had almost no effect on the evaluations. Therefore, according to findings in previous research, in the context of sponsorship-linked marketing, if the sponsored event had already occurred in the past, the information about the sponsoring brand (the actor) would play a more critical role and be given a higher weight in individuals’ evaluations than that about the sponsored event (the situation). Specifically, for the sponsored event that occurred in the past, people may pay more attention to the sponsoring brand, such as which brand sponsored the event and what it provided or contributed to the event. As people tend to focus on the limited information of the characteristics of the actor, rather than the situation in the past event, they may not care about the information of the sponsored event already having occurred. As a result, individuals’ evaluation on past sponsorship-linked marketing may be primarily determined by the data from the sponsoring brand. However, the information regarding the sponsored event would have relatively less influence. Thus, this research infers that people rely more on information regarding the sponsoring brand to evaluate the sponsorship-linked marketing campaign that occurred in the past.

Collectively, the research anticipated that temporal framing (future vs. past) and information types (the sponsoring brand and the sponsored event) jointly impact individuals’ evaluation of sponsorship-linked marketing campaigns. Specifically, as individuals require more comprehensive information to evaluate upcoming campaigns (future frame), their evaluations tend to be more positive when provided with information, both regarding the sponsoring brand and sponsored event. However, as individuals tend to focus more on the actor (the sponsoring brand) of the sponsorship that has occurred (past frame), providing information about the sponsoring brand yields more positive evaluations toward a past sponsorship campaign. In summary, the research proposes the following hypotheses:

*H1: Temporal framing and information about the sponsoring brand and sponsored event jointly influence consumers’ evaluations of the sponsorship-linked marketing campaign.*
*H1a: When the sponsorship-linked marketing campaign is framed in the future, providing information both on the sponsoring brand and sponsored event induces more positive evaluations.*
*H1b: When the sponsorship-linked marketing campaign is framed in the past, providing information on the sponsoring brand induces more positive evaluations, while information on sponsored events have less effect on evaluations.*

### Temporal Framing and Global Processing Style

The interaction between temporal framing and information type is assumedly associated with the relationship between temporal framing and processing style. Processing style refers to how an individual observes and focuses on external information, which can be general or detail-oriented (Navon, 1977; Schooler, 2002).

Global and local processing styles represent how individuals make social judgments (Förster et al., 2008). Global processing style refers to processing information from a big-picture perspective. It involves paying attention to and understanding external factors, by integrating them holistically to discover similarities. Local processing style mostly focuses on specific elements of information. Individuals with a local processing style interpret external factors by focusing on the details and identifying differences (Förster, 2009). When presented with the same event, individuals adopt either an overall or specific peripheral mindset (Liberman and Trope, 1998) and initiate global or local processing style accordingly (Förster and Dannenberg, 2010).

Temporal framing and processing styles are closely related. Previous studies show that temporal framing is associated with general and specific thinking. For example, in the context of new product evaluations, Grant and Tybout (2008) pointed out that consumers referred to comprehensive information (including both the brand and marketplace situation) when the new product launch was in the future, whereas they evaluated products primarily guided by specific information about the brand, when the launch occurred in the past. Van Boven et al. (2009) showed that the degree to which participants identified actions abstractly decreased as the temporal frame moved from the future to the past, and implied that people retrospect on past events at lower detailed levels of identification compared to prospection. Weick (1979) research on the descriptions of a car accident is also consistent with these viewpoints.

In addition, from a cognitive perspective, Zhao et al. (2014) found abstract descriptions to be more beneficial in increasing consumers’ evaluations of anticipatory advertisements, while detailed descriptions were more useful for retrospective advertisements. Further, in the variety-seeking domain, Zhang and Guo (2019) highlighted that people preferred familiarity and chose items in a narrower set when they were thinking of the past event, whereas they sought novelty and identified products in a broader set when they thought of a future event. Similar to the communication domain, Weingarten and Berger (2017) showed that individuals preferred talking more about information when the event is to happen in the future (vs. the past) and topics reflect well on the sharer. This research provides indirect evidence of the relationship between temporal framing and processing style. Therefore, future-framed events are more likely to trigger general thinking, thereby activating the global processing style, while past-framed events tend to trigger concrete thinking associated with local processing style, which then prevents global processing style from processing information. Furthermore, Pennington and Roese (2003) found that, relative to the past frame, the future frame is more likely to be associated with individuals’ promotion focus. Meanwhile, Förster and Higgins (2005) measured participants’ long-term regulatory focus tendencies in promotion and prevention orientations, requiring them to perform the Navon task, and showed a significant positive correlation between promotion focus and global processing style. Thus, relative to past-framed events, future-framed events are more likely to promote the adoption of a global processing style.

Moreover, neurological studies on cranial nerves indirectly support the relationship between temporal framing and processing style. Studies on the brain’s neural mechanisms reveal that temporal framing and processing style activate the same regions of the brain (Addis et al., 2007; Förster, 2011). Addis et al. (2007) performed brain imaging on sixteen participants using functional magnetic resonance imaging while they described past and future events. They found that participants’ left (right) hippocampus tended to activate when describing past (future) events. There was also activity in the right lateral prefrontal cortex. Other studies also found that when participants’ right brain hemisphere was activated, the effect of global processing was enhanced (Tucker and Williamson, 1984; Derryberry and Tucker, 1994; Mihov et al., 2010). Through the line bisection task, Förster (2011) and Förster et al. (2008) found that global (local) processing was more associated with right (left) hemisphere activity. As the nerve conduction velocity in the same hemisphere of the brain is higher (Gazzaniga et al., 2013), temporal framing is likely to affect the selection of processing styles. Therefore, relative to events in the past, future events are more likely to trigger the adoption of a global processing style.

In summary, this research infers that individuals who are engaged in general thinking when evaluating sponsorship-linked marketing campaigns in the future (future frame), adopt a global processing style. However, when evaluating past sponsorship-linked marketing campaigns (past frame), individuals tend to engage in concrete thinking, hindering the adoption of a global processing style. Hence, the likelihood of adopting a global processing style is reduced. Thus, the research proposes the following hypothesis:

*H2: Relative to past campaigns, future sponsorship-linked marketing campaigns are more likely to trigger the adoption of the global processing style.*

### Global Processing Style as the Underlying Mechanism

Based on the above discussion, this research posits that, when presented with campaigns within different temporal frames, the global processing style triggered by temporal framing influences the type of sponsorship information people focus on. According to Förster (2011), when applying global (local) processing style, individuals tend to adopt convergent (divergent) thinking to seek (discover) similarities (differences) across (between) objects. This notion is justified by the fact that global (local) processing and broad (narrow) conceptual domains are associated with the acceptance (rejection) of information (Schwarz and Bless, 1992, 2007; Förster, 2011; Liu et al., 2018). When mental categories are broad and inclusive, atypical category members are also accepted (e.g., individuals may consider sponsoring brands and sponsored events as belonging within the same category of business), focusing on similarities across members. When mental categories are exclusive, marginal members are rejected (e.g., individuals may consider the sponsorship as the brand’s business strategy, whereas sponsored events become benefit-irrelevant), focusing on the differences between members (Förster, 2011). For example, Friedman et al. (2003) pointed out that when the global processing style was activated, participants provided more unusual examples, including peripheral members (e.g., a camel), for a broad category (e.g., vehicles). Therefore, relative to local processing style, individuals adopting the global processing style are more likely to incorporate broader contents into information processing in evaluations.

Other studies reveal similar effects. For example, when asked to prepare for an upcoming trip, participants included more items from the same category (Liberman et al., 2002). Furthermore, the global processing style led to the inclusion of more valuable features when making judgments about strangers (Förster et al., 2009) and tolerance toward dissimilar opinions (Tuk et al., 2019). Moreover, Kühnen and Oyserman (2002) argue that a global processing style is closely associated with the interdependent self-construal, which increases the association between objects when such a style is applied. Hence, a global processing style may affect evaluations of sponsorship-linked marketing campaigns.

Research in the sponsorship-linked marketing domain show that people present higher evaluations when the images of the sponsoring brand and sponsored event are similar or consistent (Pappu and Cornwell, 2014; Kim et al., 2015; Dos Santos et al., 2019). Specifically, when evaluating upcoming campaigns (future frame), individuals are more likely to adopt a global processing style, consider the sponsoring brand and sponsored event holistically, and seek their similarities. In such cases, providing information about both the sponsoring brand and sponsored event helps individuals identify similarities and consistencies between them, inducing a more positive evaluation of the sponsorship-linked marketing. Moreover, according to the inclusion-exclusion model (Schwarz and Bless, 1992) and impression formation model (Fiske and Neuberg, 1990), as imaging a future event is an innovative experience, individuals’ considerations influence the size of expectations and additional information needs to be reinforced. Thus, providing information on sponsoring brands and sponsored events benefits the formation of positive evaluations. Finally, as the global processing style improves creativity (Friedman et al., 2003), individuals may connect and integrate the sponsoring brand and the sponsored event; hence, providing both information types may induce a more positive evaluation.

However, when evaluating past campaigns (past frame), individuals are less likely to adopt a global processing style and more likely to identify differences between the sponsoring brand and the sponsored event. Meanwhile, the data set considered becomes narrower (Förster, 2011). As individuals pay more attention to the actor in an action when evaluating past events (Jones and Harris, 1967; Gilbert et al., 1988; Gilbert and Malone, 1995; Ross et al., 2005), information on sponsored events may be considered as background or contextual information and are, therefore, excluded from evaluation processes (Friedman et al., 2003). As a result, if specific information is provided, such as that related to the sponsoring brand, it may induce a more positive evaluation. In summary, if the temporal framing effect is triggered by the adoption of the global processing style, when the local processing style is activated, the temporal framing effect likely weakens or attenuates. Hence, this research proposed the following hypotheses:

*H3a: Compared to past sponsorship-linked marketing campaigns (past frame), future campaigns (future frame) are more likely to activate a global processing style; hence, providing information on the sponsoring brand and sponsored event induces a more positive evaluation.*
*H3b: When a local processing style is activated, regardless of the temporal frame (past or future) of the sponsorship-linked marketing campaign, providing information on the sponsoring brand and sponsored event has less impact on the evaluation.*

## Study 1: The Effect of Temporal Framing and Information Type on Consumers’ Evaluation

### Method

Study 1 examined the influence of temporal framing and information type on the evaluations of sponsorship-linked marketing campaigns (H1, H1a, and H1b) in a 2 (temporal framing: 2 months ago, vs. in 2 months) × 2 (brand information: yes vs. no) × 2 (event information: yes vs. no) between-subject design. A fictitious event, “Chinese Table Tennis League,” was the sponsored event in this study; the sponsoring brand was “Tea and Painting,” a real-world brand with low brand awareness. If a future frame impels individuals’ attention toward the sponsoring brand and sponsored event, providing both sets of information would have induced a more positive evaluation. If a past frame impels individuals’ attention toward only the sponsoring brand, providing only the sponsoring brand information would have induced a more positive evaluation.

Notably, prior studies have found that the match between temporal frames and information construction affect processing fluency, which in turn affects individuals’ evaluations (Hyejeung and Schwarz, 2006; Kim et al., 2009; Zhao et al., 2014). Moreover, future-framed information increases perceived uncertainty and triggers information seeking to reduce uncertainty (Grant and Tybout, 2008). These factors may have influenced the temporal effect in the current research. Study 1 also investigated whether processing fluency or perceived uncertainty could explain the proposed effects.

### Participants and Procedure

Participants were recruited from the Sojump online survey platform and randomly assigned to one of the eight experimental conditions. Further, to ensure the seriousness and engagement of participants during the survey, minimum and maximum time limits for completing the questionnaire were defined and trick questions were included to screen participants. A prize draw was provided as an incentive to participate. In total, 440 participants were recruited. Of these, fourteen were excluded from the analysis owing to duplicate IP addresses. Ultimately, the data gathered from 426 participants (209 males; *M*_age_ = 31.48 years, *SD* = 7.56) were included in the analysis.

First, participants read the instruction, which framed the study as “a survey of a sponsorship from a brand.” On a subsequent webpage, participants were required to rate brand familiarity on a seven-point scale (1 = not at all familiar, 7 = extremely familiar), aiming to examine participants’ familiarity with the brand stimulus used in Study 1. Three brands (Anta, China Life Insurance, and Mengniu), which sponsored activities over the past year, were selected from a Chinese sponsorship website and presented together with the stimulus brand (Tea and Painting), to minimize participants’ likelihood of guessing the purpose.

On the next page, participants were provided with the news about “Tea and Painting sponsoring the Chinese Table Tennis League,” in which temporal framing, the sponsoring brand information, and the sponsored event information were manipulated. For manipulation of the temporal frames, the news’ headline presented to the future-framed group was: “Tea and Painting will sponsor the Chinese Table Tennis League in 2 months,” while that presented to the past-framed group was: “Tea and Painting sponsored the Chinese Table Tennis League 2 months ago.” At the same time, temporal frames were repeated in the main body of the news accordingly. For manipulation of the sponsoring brand information, the group that was provided with a brand introduction of Tea and Painting could read information on brand positioning, philosophy, and achievements; while the no-sponsoring-brand-information group would not read such instructions. Similarly, for manipulation of the sponsored event information, the sponsored-event-information group was provided with an introduction to the Chinese Table Tennis League, including the status and commercial value, while the no-sponsored-event-information group would not read these instructions. The length of the news introducing the brand and event information were similar at approximately 110 words per piece. A minimum time spent on the page was set at 30 s to ensure participants fully read the information.

Next, participants were required to evaluate the (potential) effectiveness of the sponsorship-linked marketing campaign. According to Kim et al. (2009), participants were asked to make evaluations on six seven-point scales items (Cronbach’s α = 0.880), from the perspective of the sponsored event and the news article, the former (4 items) modified following Ruth and Simonin (2003) and the latter (2 items) adapted from Kim et al. (2009) (see Table 1 for details of the measures).

**TABLE 1:** Measurements: items, reliabilities and validities (Study 1 and Study 3).

Measurements and items	Study 1			Study 3		
	CA	CR	AVE	CA	CR	AVE
Evaluation of the sponsorship-linked marketing event						
Strongly dislike/strongly like	0.880	0.894	0.585	0.841	0.845	0.478
Extremely unpleasant/extremely pleasant						
Extremely unenjoyable/extremely enjoyable						
Extremely poor/extremely good						
Extremely unimpressive/extremely impressive						
Extremely unimportant/extremely important						
Processing fluency						
Extremely uncomfortable/extremely comfortable	0.899	0.900	0.752			
Extremely unattractive/extremely attractive						
Extremely unpersuasive/extremely persuasive						
Perceived uncertainty (*strongly disagree/strongly agree*)						
I feel a sense of uncertainty toward this sponsored event.	*0.735^a^*	0.868	0.770			
I think this sponsored event is risky.						
Brand familiarity						
I never heard of it/I hear it frequently				0.759	0.761	0.516
I do not know it at all/I know it very well						
Extremely unfamiliar/extremely familiar						

*CA, Cronbach’s Alpha; CR, Composite Reliability; AVE, Average Variance Extracted; ^a^The related coefficient was used in Perceived uncertainty, because there were only two items in this measurement.*

On the next webpage, two alternative explanations (processing fluency and perceived uncertainty) were measured. Specifically, Study 1 assessed processing fluency with three seven-point scales items (Cronbach’s α = 0.899) from Hyejeung and Schwarz (2006). For assessing perceived uncertainty, participants were required to indicate their agreement with two sentences (1 = strongly disagree, 7 = strongly agree; γ = 0.735; *p* < 0.001) (see Table 1 for details for the two measures).

Then, participants indicated their perceptions of time. For temporal framing, they were asked to select the time point of the sponsorship event on a seven-point scale (1 = occurred in the past, 7 = will occur in the future) (Zhao et al., 2014). For temporal distance, they were asked how close to the present day the sponsorship seemed on a seven-point scale, ranging from relatively close (1) to relatively far (7) (Grant and Tybout, 2008).

Finally, participants provided demographic information (e.g., gender and age). After submitting the questionnaire, participants were provided a link to participate in the prize draw.

### Results and Discussion

#### Manipulation Check

First, temporal framing and temporal distance were checked.

A 2 (temporal framing) × 2 (sponsoring brand information) × 2 (sponsored event information) between-subjects ANOVA was conducted using temporal framing, sponsoring brand information, sponsored event information as fixed factors, and perceived time points as the dependent variable. Accordingly, the main effect of temporal framing on the perceived time point was significant (*F* [1, 418] = 633.918, *p* < 0.001, η_*p*_^2^ = 0.599). Relative to the future-frame group (*M*_*two months later*_ = 5.472, *SD* = 1.086), the past-frame group noticeably differed in perceiving the sponsored event in the past (*M*_*two months ago*_ = 2.944, *SD* = 0.977); however, no other effect was significant (*ps* > 0.3, η_*p*_^2^ *s* < 0.004; see Table 2).

**TABLE 2:** Means and standard deviations for variables and manipulation checks (Study 1–Study 3).

Studies	Evaluation of the sponsorship-linked marketing event				Figure identification score	Determining basis		Perceived time point	Perceived temporal distance
**Study 1**	*Information on the sponsored event*								
	
	yes		no						

Temporal framing	*Information on the sponsoring brand*		*Information on the sponsoring brand*						
	
	yes	no	yes	no					

Future framing	5.063 (1.182)	4.903 (1.122)	5.044 (1.021)	3.299 (1.100)				5.472 (1.086)	5.212 (1.348)
Past framing	5.059 (0.948)	3.928 (1.368)	4.744 (1.189)	4.044 (1.178)				2.944 (0.977)	5.047 (1.443)

**Study 2**						*Overall shapes*	*Smaller shapes*		

Temporal framing									
Future framing					11.160 (3.260)	4.640 (1.816)	3.980 (1.708)	5.460 (1.297)	5.480 (1.297)
Past framing					9.600 (2.711)	3.880 (1.423)	4.800 (1.457)	3.260 (1.322)	5.120 (1.380)

**Study 3**									

Temporal framing	*Global processing style*		*Local processing style*						

Future framing	5.507 (0.791)		5.104 (0.852)					5.222 (1.036)	5.220 (1.107)

Past framing	5.134 (0.879)		5.310 (0.687)					2.685 (1.104)	5.154 (1.159)

**Manipulation check of processing style for Study 3**						*Overall shapes*	*Smaller shapes*		

Processing style									
Global processing style					12.250 (1.446)	4.650 (1.040)	3.900 (1.021)		
Local processing style					10.550 (1.905)	3.850 (1.137)	4.800 (1.196)		

*Standard deviations in parentheses.*

A 2 × 2 × 2 between-subjects ANOVA was then conducted, using perceived temporal distance as the dependent variable. The results showed no significant difference in the perceived temporal distance between two groups (*F* [1, 418] = 1.497, *p* = 0.222, η_*p*_^2^ = 0.003); no other effect was significant (*ps* > 0.2, η_*p*_^2^ *s* < 0.003; see Table 2). In sum, the manipulation of temporal framing met expectations.

Second, brand familiarity was checked.

The results from the descriptive analysis of four brands’ familiarity showed that participants felt less familiarity with Tea and Painting than the median value (*M* = 2.707, *SD* = 1.074) and that of the other brands (*M*_*Anta*_ = 5.336, *SD* = 1.428; *M*_*China Life Insurance*_ = 5.345, *SD* = 1.418; and *M_*Mengniu*_* = 5.380, *SD* = 1.339).

A 2 × 2 × 2 between-subjects ANOVA was then conducted, using familiarity of Tea and Painting as the dependent variable. The results showed no significant main effect (*F* [1, 418] < 2, *ps* > 0.2, η_*p*_^2^ *s* < 0.003); the interaction effect of three factors was also not significant (*F* [1, 418] = 0.154, *p* = 0.695, η_*p*_^2^ = 0.000); no other significant effect was found (*ps* > 0.1, η_*p*_^2^ *s* < 0.002). Therefore, the effects in Study 1 were not associated with brand familiarity.

#### Hypotheses Testing

A 2 × 2 × 2 between-subjects ANOVA was conducted, evaluations on the sponsoring-linked marketing campaigns served as the dependent variable. The results showed that the main effect of sponsoring brand information was significant (*F* [1, 418] = 71.002, *p* < 0.001, η_*p*_^2^ = 0.145). The evaluations of participants provided with the sponsoring brand information (*M* = 4.977, *SD* = 1.090) were significantly more positive than those without the information (*M* = 4.043, *SD* = 1.319). Further, the main effect of sponsored event information was also significant (*F* [1, 418] = 16.876, *p* < 0.001, η_*p*_^2^ = 0.039). The evaluations of participants provided with the sponsored event information (*M* = 4.739, *SD* = 1.248) were significantly more positive than those without (*M* = 4.285, *SD* = 1.304). Finally, the interaction effect of temporal framing, sponsoring brand information, and sponsored event information was significant (*F* [1, 418] = 20.671, *p* < 0.001, η_*p*_^2^ = 0.047). However, the main effect of temporal framing on participants’ evaluations was not significant (*F* [1, 418] = 1.450, *p* = 0.229, η_*p*_^2^ = 0.003). Hence, H1 was supported.

Moreover, the influence of sponsoring brand and sponsored event information on evaluations in different time frames were explored, respectively.

Under the future frame (in 2 months), both the main effects of sponsoring brand information (*F* [1, 418] = 39.688, *p* < 0.001, η_*p*_^2^ = 0.159) and sponsored event information (*F* [1, 418] = 28.632, *p* < 0.001, η_*p*_^2^ = 0.120) were significant. When the brand information of Tea and Painting was provided, participants’ evaluations (*M* = 5.503, *SD* = 1.099) were significantly more positive than those of participants without the information (*M* = 4.101, *SD* = 1.368). When information on the Chinese Table Tennis League was provided, participants’ evaluations (*M* = 4.983, *SD* = 1.149) were also significantly more positive than those when the information was not provided (*M* = 4.171, *SD* = 1.372). Further, the interaction effect of sponsoring brand information and sponsored event information was significant (*F* [1, 418] = 54.881, *p* < 0.001, η_*p*_^2^ = 0.115).

**FIGURE 1:**
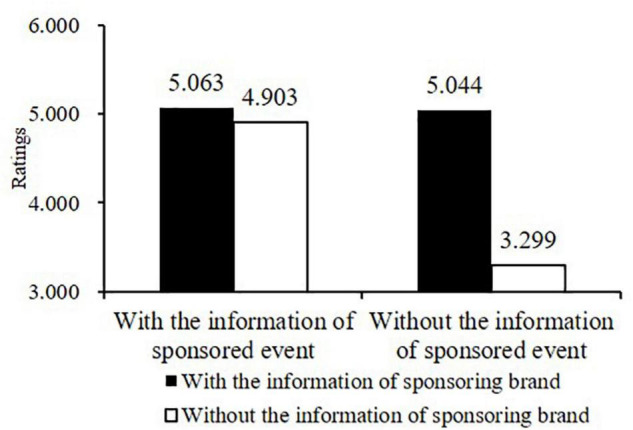
Results of Two-Way ANOVA under the Future-Frame Condition in Study 1.

Specifically, under the future frame, when provided with information on the Chinese Table Tennis League, participants’ evaluations were not affected by the sponsoring brand information (*M_*with*_* = 5.063, *SD* = 1.182; *M_*without*_* = 4.903, *SD* = 1.122; *F* [1, 418] = 0.521, *p* = 0.471, η_*p*_^2^ = 0.005). However, when information on the Chinese Table Tennis League was not provided, evaluations from participants with sponsoring brand information (*M* = 5.044, *SD* = 1.021) were significantly more positive than those without (*M* = 3.299, *SD* = 1.100; *F* [1, 418] = 61.679, *p* < 0.001, η_*p*_^2^ = 0.403). Evaluations from participants with neither the sponsoring brand nor the sponsored event information were the most negative (*M* = 3.299, *SD* = 1.100), indicating that providing at least one type of sponsorship information (the sponsoring brand or the sponsored event) could facilitate positive evaluations (see Figure 1). Hence, H1a was supported.

**FIGURE 2:**
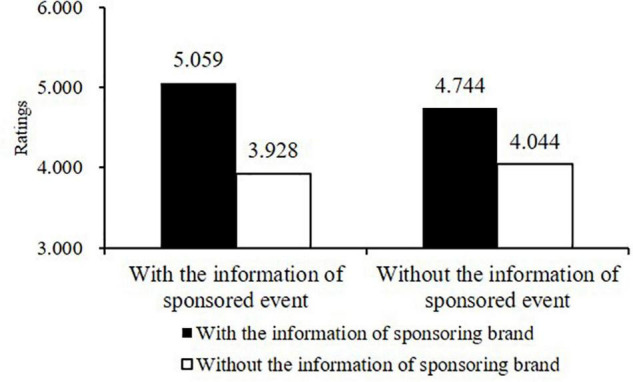
Results of the Two-Way ANOVA under the Past-Frame Condition in Study 1.

Under the past frame (2 months ago), the main effect of the sponsoring brand information was significant (*F* [1, 418] = 32.498, *p* < 0.001, η_*p*_^2^ = 0.133); however, that of the sponsored event information was not significant (*F*[1, 418] = 0.378, *p* < 0.001, η_*p*_^2^ = 0.008), and the interaction effect of the two information types was also insignificant (*F* [1, 418] = 1.779, *p* = 0.183, η_*p*_^2^ = 0.008). Only when participants were provided with the sponsoring brand information did their evaluations (*M* = 4.901, *SD* = 1.082) become significantly more positive than those without (*M* = 3.986, *SD* = 1.272) (see Figure 2). Hence, H1b was supported.

#### Alternative Explanation

The bias-corrected non-parametric percentile bootstrap test was conducted to test the significance of the mediating effects of processing fluency and perceived uncertainty (Preacher and Hayes, 2008). According to Hayes and Preacher (2014) (model 19, 5,000 Bootstraps at a 95% confidence interval), this study introduced temporal framing as the independent variable, sponsoring brand information and sponsored event information as moderating variables, campaign evaluation as the dependent variable, and processing fluency and perceived uncertainty as mediating variables (respectively) into the model. The results showed that the direct effect of the interaction of temporal framing, sponsoring brand information, and sponsored event information on the evaluation was statistically significant (*t* = −4.605, *p* < 0.001), while neither the indirect effect of processing fluency (LLCI = −0.0212, ULCI = 0.1552; including 0) nor perceived uncertainty (LLCI = −0.2345, ULCI = 0.2509; including 0) was significant. Thus, processing fluency and perceived uncertainty could not explain the influence of temporal framing, sponsoring brand information, and sponsored event information on the evaluations of the sponsoring-linked marketing.

#### Discussion

Study 1 revealed that when the sponsoring-linked marketing campaign would take place in 2 months (future frame), providing participants with information on the sponsoring brand and sponsored event resulted in their evaluations being more positive; however, when the campaign took place 2 months ago, only when the sponsoring brand information was provided, were participant evaluations more positive, while providing the sponsored event information had no effect on evaluations. Therefore, the findings support H1, H1a, and H1b. Specifically, when a sponsorship-linked marketing campaign occurs in the future, people are more likely to pay attention to the sponsored brand and sponsoring event (H1a). However, when the campaign was in the past, people would focus more on the sponsoring brand (H1b). Thus, temporal framing causes people to pay attention to different types of information which then affects their evaluations on sponsorship-linked marketing campaigns. The results also demonstrate that either processing fluency or perceived uncertainty could explain the interaction effect of temporal framing and information type on individuals’ evaluations.

The present research argues that temporal framing affects the processing style adopted to process received information, which induces people to focus on different information types under different temporal frames. Next, Study 2 investigates the relationship between temporal framing and processing style (H2), while Study 3 examines whether processing style explains the influence of temporal framing and information type on evaluations (H3a and H3b).

## Study 2: Temporal Framing and Processing Style

### Method

Study 2 applied a single factor (temporal framing: 3 weeks ago vs. in 3 weeks) between-subjects design. According to Kim et al. (2009), the scenario in Study 2 involved a fictional brand (Deral) sponsoring the event “Original Music Contest on Campus.” The brand name was created based on the Italian sports brand Diadora to avoid the influence of participants’ prior experience with the actual brand on their evaluations. To enhance involvement, a familiar event for college students could improve the relevance between the participants and sponsored event. The study implied that, relative to the past-framed group, the future-framed group would be more likely to adopt a global processing style.

### Participants and Procedure

One hundred undergraduates and graduate students were recruited (forty-nine males; *M*_age_ = 21.61 years, *SD* = 2.54) and randomly assigned to one of the two experimental conditions (past and future). Each participant who completed the task was provided a pen as compensation.

Participants first read the instructions, as in Study 1, and were required to read the following news carefully, imagine the sponsorship-linked marketing event, and provide a summary description.

Then, a news headline titled: “Deral Sponsors the Original Music Contest on Campus” was presented. Only the temporal frame was manipulated in the news. The future-framed group was presented with: “Deral will sponsor the Original Music Contest on Campus in 3 weeks,” while the past-framed group received: “Deral sponsored the Original Music Contest on Campus 3 weeks ago.” Further, the temporal frames were reinforced in the main body of the news. The length of the texts presented to the two groups was similar at approximately 110 words per piece. After reading the news, participants were required to spend some time imagining and describing the event without a time limit. Open questions were used to allow participants to engage more in the scenario; however, their descriptions were not used in the subsequent data analysis. The measures of perceived time point and temporal distance were the same as in Study 1.

**FIGURE 3:**
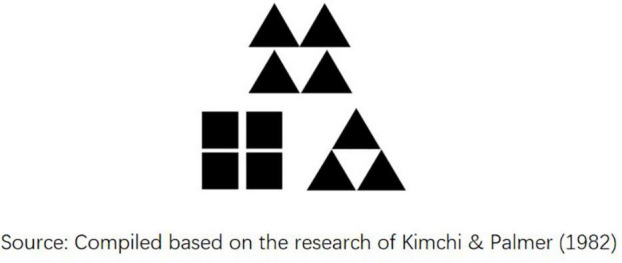
Example Figures in the Figure Identification Task in Study 2.

Next, participants’ tendencies to adopt a global processing style were measured. Specifically, following the procedure from Kimchi and Palmer (1982), participants completed a figure identification task which was used to explore whether positive emotions led to a global processing style (Gasper and Clore, 2002). The task used attentional bias to determine the processing style adopted, which included sixteen sets of subtasks. Each set included three entire figures (large triangles or squares) composed of small figures (small triangles or squares). The standard figure was placed at the top, and the two comparison figures were placed below (e.g., in Figure 3). One of the comparison figures matched the entire shape of the standard figure, while specific parts of the other comprised figures matched the smaller shapes in the standard figure. Participants were required to determine which of the comparison figures was most like the standard figure. If one chose the more general comparison figure, they adopted a global processing style (global attentional bias). If one selected the figure with more specific comparisons, then they adopted a local processing style (local attentional bias). Participants were only permitted to select one of the comparison shapes for each set. After completing the sixteen sets of subtasks, they provided ratings on two seven-point scale items (1 = totally disagree, 7 = totally agree) regarding how much they agreed with the following two statements: “I made the choice mainly based on the overall similarity of the two figures. For example, a large square made up of small triangles is more like a large square made up of small squares” (the basis to confirm a global processing style) and “I made the choice mainly based on the small shapes that composed the two figures. For example, a large square made up of small triangles is more like a large triangle made up of small triangles” (the basis to confirm a local processing style).

Finally, participants provided demographic information and received a small gift for participation.

### Results and Discussion

#### Manipulation Check

The results from the independent sample *t*-test showed that the perceived time point was significantly different between the two groups (*t* [98] = −8.400, *p* < 0.001, η_*p*_^2^ = 0.414). Relative to the future-frame group (*M_*three weeks later*_* = 5.460, *SD* = 1.297), the past-frame group significantly differed in their perceptions of sponsored events in the past (*M_*three weeks ago*_* = 3.260, *SD* = 1.322). Moreover, no significant difference was found in the perceived temporal distance between the two groups (*t* [98] = −1.344, *p* > 0.1, η_*p*_^2^ *s* = 0.018; see Table 2). Hence, the manipulation of temporal framing met expectations.

#### Hypotheses Testing

First, selected figures from the figure identification task were coded and rated. Specifically, if the participant selected the figure that matched the entire shape of the standard figure, it was rated as 1; otherwise, it was rated as 0. For example, if the standard figure was a large square composed of four small triangles, the first comparison figure (on the left) may have been a large square composed of four smaller squares with the same entire shape as the standard figure. Hence, selecting the left figure was coded as 1. The other comparison figure (on the right) was a large triangle composed of three smaller triangles, which were the same shape as part of the standard figure, and the selection of this was rated as 0. The total scores of the 16 sets were summed to obtain the processing style score for each participant. The higher the score, the greater the tendency to adopt a global processing style.

Next, the results from the independent sample *t*-test, using the figure identification score as the dependent variable, revealed a significant main effect of temporal framing (*t* [98] = −2.602, *p* = 0.011, η_*p*_^2^ = 0.065). Relative to the past-frame group, the future-frame group obtained significantly higher scores (*M_*in three weeks*_* = 11.160, *SD* = 3.260; *M_*three weeks ago*_* = 9.600, *SD* = 2.711; see Table 2). Moreover, the results from the independent sample *t*-test, using determining basis as the dependent variable, also revealed significant main effects of temporal framing. Specifically, as shown in Table 2 relative to the past-frame group, the future-frame group was more inclined to determine the similarity of the entire shapes of the figures (*M_*three weeks ago*_* = 3.880, *SD* = 1.423; *M_*in three*__*weeks*_* = 4.640, *SD* = 1.816; t[98] = −2.329, *p* = 0.022, η_*p*_^2^ = 0.053) and less likely to identify the similarity of the smaller shapes comprising the figures (*M_*three weeks ago*_* = 4.800, *SD* = 1.457; *M*_*in three weeks*_ = 3.980, *SD* = 1.708; t[98] = 2.583, *p* = 0.011, η_*p*_^2^ = 0.064). These results showed that temporal framing triggered processing styles, and under the future frame, participants were more likely to adopt a global processing style. Hence, H2 was supported.

#### Discussion

Study 2 revealed that, relative to participants who read the news of a sponsorship-linked marketing campaign having occurred 3 weeks ago, those who read the news of an event 3 weeks later, were more inclined to adopt a global processing style when making judgments. In other words, relative to the past-framed group, the future-framed group was more likely to adopt a global processing style when processing information.

Next, Study 3 examined the underlying mechanism of the global processing style’s role in the effect of temporal framing and information type on the evaluations (H3a and H3b). Notably, to verify the underlying mechanism of the global processing style more rigorously and effectively, instead of rating the processing style (as in Study 2) and conducting a mediation analysis, as recommended by Baron and Kenny (1986), Study 3 manipulated the processing style. Adopting such an approach was based on the following three considerations. First, as the task in Study 2 comprised sixteen sets of subtasks, requiring much time and energy, participants may have been hesitant to continue through the subsequent tasks and may have been unduly concerned with how they were required to participate. Second, in other studies, processing style is usually activated via visual discrimination tasks (Navon, 1977) and abstract-concrete word-judgment tasks (Förster et al., 2008; McCrea et al., 2012). Due to the requirement of a laboratory setting in the Navon task (Navon, 1977), Study 3 employed an abstract-concrete word-judgment task (Förster et al., 2008; McCrea et al., 2012), which was commonly employed in behavioral research to prime global-local processing style. Third, manipulating the mediator could have avoided low discriminant validity caused by the reliability of measures (Spencer et al., 2005).

## Study 3: The Underlying Mechanism of Global Processing Style

### Method

Study 3 applied a 2 (temporal framing: 2 weeks ago vs. in 2 weeks) × 2 (processing style: global vs. local) between-subjects design. The materials used in Study 3 were modified based on Study 2. The sponsorship was changed to: “Deral sponsors the China Original Singer Music Festival,” and the temporal framing was changed to “2 weeks ago” (past frame) and “in 2 weeks” (future frame). The adoption of a shorter temporal distance (relative to that used in the previous two studies of 2 months, and 3 weeks) aimed to test the robustness of the results. This study followed Grant and Tybout (2008) on manipulating information type to simplify the explanation of the hypotheses. Information on the sponsoring brand and sponsored event was kept the same for the conditions. Specifically, only the sponsored event information was provided, and the description of the sponsoring brand was brief (including only the origin nation and target market) to avoid the influence of other information on the interpretation of results. The study implied that if a future frame stimulates the adoption of a global processing style, participants would require information on the sponsoring brand and sponsored event for their evaluations. Hence, when local processing style is primed, any temporal framing effect likely weakens or attenuates, that is, the difference in positive evaluations between future- and past-framed groups should decrease or disappear.

### Participants and Procedure

The participants consisted of 179 undergraduates and graduate students (91 males; *M*_age_ = 21.27 years, *SD* = 2.48) randomly assigned to one of four experimental conditions. A gift was provided as the compensation.

First, participants read the instructions, which introduced the study as two unrelated tasks: describing daily activities and an investigation of the effectiveness of a sponsored event.

In the first task, a global-local processing style was manipulated. According to Förster et al. (2008) and McCrea et al. (2012), the abstract-concrete word-judgment task was applied to activate the processing styles. Participants were required to describe ten daily activities (writing a diary, calling a high-school classmate, participating in a sport, painting a dormitory, washing hands, revising class materials, playing the piano, surfing the Internet, reading a book, and helping a friend move to a new apartment). Participants in the global-processing style group were required to write two adjectives to describe the characteristics of each activity, while those in the local-processing style group were required to write two items involved in completing each activity.

After completing the description task, in the second task, participants were presented with news: “Deral is sponsoring the Chinese Original Singer Music Festival.” Temporal framing was manipulated in the news headline. For the future-framed group, the headline was: “The Chinese Original Singer Music Festival will open in 2 Weeks,” while that for the past-framed group was: “The Chinese Original Singer Music Festival Opened 2 Weeks Ago.” Moreover, the temporal framings were repeated in the main body of the news report accordingly. There was also an introduction of the China Original Singer Music Festival in the news, including its development and influence. The lengths of the two pieces were similar at approximately 240 words per piece.

After reading the news, participants evaluated the campaign on six items, as in Study 1 (Cronbach’s α = 0.841). Also, they rated perceived time points and temporal distance on items, as in Study 1. Then, participants indicated their familiarity toward the brand Deral, on three seven-point scales (see Table 1 for details; Cronbach’s α = 0.759).

Finally, participants provided demographic information and received a gift as compensation.

### Results and Discussion

#### Manipulation Check

First, 40 college students (19 males; *M*_age_ = 21.68 years, *SD* = 2.51) from the same subject pool that had not participated in Study 3 were asked to complete the manipulation check of processing style. They were randomly assigned to one of the two conditions (global vs. local processing style). After completing the description of 10 daily activities (McCrea et al., 2012), they were required to undertake the figure identification task (Kimchi and Palmer, 1982) and rate the determination basis items used in Study 2. The results of the independent sample *t*-test showed that the main effect of processing style was significant. Specifically, relative to students that were asked to provide a list of items used in daily activities (local processing style), those that were asked to provide adjectives to describe the activities (global processing style) scored significantly higher in the figure identification task (*M_*item*_* = 10.550, *SD* = 1.905; *M_*adjective*_* = 12.250, *SD* = 1.446; *t_*figure*__*recognition*_* [38] = −3.179, *p* = 0.003, η_*p*_^2^ = 0.210; see Table 2). They were also more inclined to adopt a global processing style to determine the similarity of the figures (*M*_*item*_ = 3.850, *SD* = 1.137; *M_*adjective*_* = 4.650, *SD* = 1.040; *t_*global*_*[38] = −2.322, *p* = 0.026, η_*p*_^2^ = 0.124) and less inclined to adopt a local processing style (*M*_*item*_ = 4.800, *SD* = 1.196; *M_*adjective*_* = 3.900, *SD* = 1.021; *t*_*local*_[38] = 2.559, *p* = 0.015, η_*p*_^2^ = 0.147; see Table 2). Hence, the abstract-concrete word-judgment task effectively activated global and local processing styles, respectively.

Second, a 2 (temporal framing) × 2 (processing style) two-factor ANOVA was conducted; perceived time point and perceived temporal distance served as dependent variables, respectively. The results showed that the main effect of temporal framing on perceived time point was significant (*F* [1, 175] = 250.745, *p* < 0.001, η_*p*_^2^ = 0.586). Relative to the future-frame group (*M_*two weeks later*_* = 5.222, *SD* = 1.036), the past-frame-group was noticeably more aware that the sponsored event occurred in the past (*M_*two weeks ago*_* = 2.685, *SD* = 1.104); no other effect was significant (*ps* > 0.3, η_*p*_^2^ *s* < 0.003). Moreover, there was no significant difference in the perceived temporal distance between the two groups (*F* [1, 175] = 1.917, *p* = 0.168), and other effects were not statistically significant (*ps* > 0.5, η_*p*_^2^ *s* < 0.001). Thus, the manipulation of temporal framing met expectations.

Third, the effect of brand familiarity was examined. The results of descriptive statistics revealed that participants’ familiarity with the brand was lower than the median (*M* = 2.575, *SD* = 0.844); that is, participants were not familiar with the fictional brand in the materials. Further, 2 × 2 between-subject ANOVA on brand familiarity found no significant effect (*p* > 0.7, η_*p*_^2^ *s* < 0.001). Hence, the effects observed in Study 3 were not associated with brand familiarity.

#### Hypotheses Testing

The results from the 2 × 2 between-subject ANOVA, using the evaluation as a dependent variable, showed that the main effect of either temporal framing (*F* [1, 175] = 0.478, *p* = 0.490, η_*p*_^2^ = 0.003) or processing style (*F* [1, 175] = 0.890, *p* = 0.347, η_*p*_^2^ = 0.005) was also not significant; however, the interaction effect of temporal framing and processing style was significant (*F* [1, 175] = 5.767, *p* = 0.017, η_*p*_^2^ = 0.032).

**FIGURE 4:**
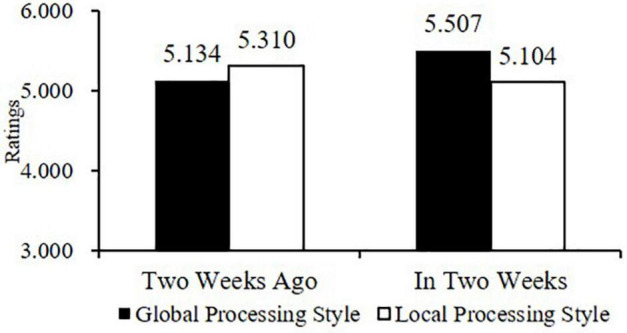
Results of the Two-Way ANOVA in Study 3.

When global processing style was primed, the evaluation from the future-frame group (*M*_*in two weeks*_ = 5.507, *SD* = 0.791) was significantly more positive than that from the past-frame group (*M_*two weeks ago*_* = 5.134, *SD* = 0.879; *F* [1, 175] = 4.866, *p* = 0.029, η_*p*_^2^ = 0.027). Hence, H3a was supported. Moreover, when local processing style was primed, the evaluations from the two groups had no significant difference (*M_*two weeks ago*_* = 5.310, *SD* = 0.687; *M*_*in two weeks*_ = 5.104, *SD* = 0.852; *F* [1, 175] = 1.437, *p* = 0.232, η_*p*_^2^ = 0.008). Thus, H3b was supported (see Figure 4).

As all participants in Study 3 were provided with information about the sponsored event, when global processing style was primed, relative to the past-frame group that evaluated the campaign based on the sponsoring brand information, the future-frame group also evaluated the campaign based on the information about the sponsored event. Hence, a temporal framing effect remained. However, when local processing style was primed, the expectations of the future-frame group for more comprehensive information were reduced, and their attention to specific brand information increased, resulting in less differences in evaluations between the future- and the past-frame groups. In sum, due to the relationship between temporal framing and processing style subsequently activated, there were different evaluations between people under different temporal frames.

#### Discussion

Study 3 confirmed that the processing style affected participants’ evaluations of sponsorship-linked marketing campaigns under different temporal frames. Specifically, when a global processing style was primed and only sponsored event information was provided, the future-framed group tended to evaluate the campaign more positively than the past-framed group. However, if a local processing style was primed, the global processing style (usually applied to future events) was inhibited and as a result, the future-framed group paid less attention to the sponsored event information. Thus, their evaluations of the campaign showed no significant difference to those of the past-framed group. The results of Study 3 supported the hypothesis that global processing style served as the underlying mechanism.

## General Discussion

### Conclusion

From the perspective of external temporal cues, based on theories of temporal framing and processing style, the current research explored the effect of temporal framing and information type on individuals’ evaluations of sponsorship-linked marketing campaigns. It is noteworthy that diverse types of sponsorship information had various weights on the evaluations under different frames of time without the influence of the perceived distance from the sponsorship campaign. Specifically, the sponsoring brand information was preferred more with past framing; however, both the information of the sponsoring brand and the sponsored event were considered in future framing. Additionally, discovering a close relationship between temporal framing and global processing style was also interesting. People in future framing were more likely to adopt the global processing style when they made evaluations.

Study 1 recruited participants on an online platform to investigate the interaction effect involving a real brand and event. The results indicated that when the sponsored China Table Tennis was to occur in 2 months (future framing), participants who were presented with information about both the sponsoring brand, namely Tea and Painting, and the sponsored event, evaluated the campaign more positively; however, when the sport event occurred 2 months ago (past framing), only presenting the information about the sponsoring brand, Tea and Painting, increased participants’ evaluations of the campaign, and providing the information about the sport event did not affect the evaluations. Study 1 also ruled out the alternative explanations of processing fluency and perceived uncertainty. Why did the information about the sponsoring brand and the sponsored event have different influences on individuals’ evaluations under different temporal framings? Study 2 and 3 explored the underlying mechanism of global processing style on the temporal effect. Study 2 first tested the relationship between temporal framing and global processing style. In the figure identification task, compared to participants who read the news of the sponsorship campaign having occurred 3 weeks ago (past framing), those who read the news of the campaign occurring in 3 weeks (future framing) obtained higher scores (indicating a general judgment), and were more likely to make evaluations on a global decision basis. Furthermore, Study 3 manipulated the processing style and presented the same information (including a particular introduction to the sponsored event and a brief description of the sponsoring brand). The results showed that when a global processing style was primed, participants who read the news of the sponsorship opening in 2 weeks (future framing) evaluated the campaign more positively than those who read a news headline from 2 weeks ago (past framing); however, if a local processing style was primed, the difference between the two temporal framing groups was attenuated. Thus, the underlying mechanism of global processing style was confirmed.

Taken together, these three studies revealed that temporal framing, as an external time cue, stimulated the adoption of a global processing style during the evaluation process. Global processing style impacted the information type on which individuals paid attention (sponsoring brand and sponsored event); that is, people screened information accordingly during the evaluation process. Relative to a past frame, the future frame increased the adoption of the global processing style, especially regarding the “big picture” understanding of the upcoming event. Therefore, providing information on the sponsoring brand and the sponsored event resulted in more positive evaluations. Past framing, however, reduced the adoption of the global processing style, which resulted in more attention being focused on the actor (the sponsoring brand), while ignoring the more general context (the sponsored event). Thus, providing information on the sponsoring brand was more likely to yield a positive evaluation, while the provision of sponsored event information appeared to have no significant impact on the evaluation. The present research employed the experimental studies of real events and fictitious scenarios, which were tested among diverse samples (college students and ordinary adults), different temporal distance settings (2 months, 3 weeks, and 2 weeks), various sponsored activities (sports and music events), dissimilar brand stimuli (real and fictional brands), and the probable explanation of the influence of processing fluency and perceived uncertainty. Therefore, the conclusions had good validity and generalizability.

The current research is distinguished from and extends prior studies in three ways. First, this research distinguished the influence of past and future frames on individuals’ evaluations of sponsorship-linked marketing campaigns, while past studies focused only on single temporal framing, that is, either the past or the future (Lee et al., 2017; Xu and Jin, 2021). Research on the planning fallacy and affective forecasting focused on events in the future, which identifies biases that impair the ability to predict one’s responses to future events (Buehler et al., 1994; Buehler and McFarland, 2001; Van Boven et al., 2009). In contrast, research on the hindsight bias and causal attribution theory considered past events. Only a few studies have examined the effect of framing the same event in the future vs. the past (Grant and Tybout, 2008). Second, this research explained the influence of temporal framing as an external time cue from the perspective of processing style, different from the perspective of individual internal perceptions used in past studies, such as a desire to savor the experience (Huang et al., 2016) and familiarity or novelty seeking (Zhang and Guo, 2019). Although many researchers suggest that time-related issues should be investigated, they remain largely ignored in empirical research (Lin and Liao, 2020; Xi et al., 2021). Third, but most important, the current research also investigated whether the manipulations of temporal framing generated differences in the perceived temporal distance from the sponsorship campaigns. Previous research has revealed that the perceived distance from an event (near or far) influenced the construal level (Trope and Liberman, 2003). Specifically, people represented distant events more abstractly, focusing on goals and outcomes, whereas they represented near events more concretely, focusing on actions that need to happen. If the sponsorship campaign was perceived to be nearer in the future vs. the past, then a more concrete construal might result in greater attention to contextual details when people evaluated the campaigns. However, the measures of perceived distance from the sponsorship campaign showed no significant main effect of temporal framing on evaluations in Studies 1 and 3. Furthermore, the findings here were robust across three temporal intervals: 2 months (Study 1), 3 weeks (Study 2), and 2 weeks (Study 3).

### Theoretical Contributions

*First, the current research compared the differences between past and future frames and provided a new research perspective on the contextual priming of temporal framing.* Past studies on temporal framing mostly consider it to be a stable personality trait and generally discuss individuals’ attitudes toward information (Zhao et al., 2014), action plans (Rojas-Méndez and Davies, 2005), and decision making (Li, 2008) of people with different temporal orientations, largely overlooking the temporary influence of temporal framing on individual behaviors. In addition, prior studies tend to investigate the effects of past and future framing as separate constructs, where temporal framing is seen to stem from a single direction (Li, 2008; Martin et al., 2009; Van Ittersum, 2012), using present framing as the control group (Mogilner et al., 2012). However, past and future frames have diametrically opposite directions, and their influences on individuals have not yet been fully explored and discussed. Different from previous studies, the current research indicated that past and future frames, as external time cues, led to different degrees of a global processing style, which led to different evaluations and decisions. Furthermore, under the influence of temporal framing, individual information preferences are also different (either the information of the actor, or both the information of the actor and the background). The comparative exploration in this research highlights the theoretical need for more in-depth research on temporal framing.

*Second, applying temporal framing as an external time cue, this current research was one of the first to explore the relationship between temporal framing and information processing style.* It found that relative to news headlines on past sponsorships, future sponsorship news headlines triggered the adoption of the global processing style. In the domain of cognitive processing, past studies on temporal framing mainly examined its impact as a functional factor on individuals’ attitudes toward information (Zhao et al., 2014) and the need for information (Grant and Tybout, 2008). However, the driving role of temporal framing in information processing has been generally overlooked. In addition, theories of temporal psychological distance typically assume, explicitly or implicitly, temporal symmetry. That is, the judgmental and behavioral effects of being temporally close to or far from an event, presented similarly, in retrospection and in prospection. However, the research reviewed here suggests that these theories are descriptively incomplete (Perunovic and Wilson, 2009; Van Boven et al., 2009).

For example, CLT reveals that people construe temporally distant events at a higher, more abstract level than temporally proximate events, and that “the same general principles hold true for other distance dimensions, including temporal distance from past events” (Trope and Liberman, 2003; Van Boven et al., 2009). However, from the processing style perspective, the major results reported here suggest that people create more degrees in a global processing style in future events than in past events. That is, the level of construal may increase more steeply when that distance increases toward the future than toward the past. Thus, this current research found that temporal framing is closely associated with a global processing style, which made a beneficial contribution to CLT and the psychological time domain.

*Finally, this present research explored the effectiveness of sponsorship-linked marketing campaigns from the perspective of temporal frames, which provided new evidence and theoretical foundations for the sponsorship-linked marketing domain.* Prior studies mostly focused on the impact of sponsorship-linked marketing on corporate financial performance (Mazodier and Rezaee, 2013), the fit between sponsoring brands and sponsored events (Pappu and Cornwell, 2014; Kim et al., 2015; Dos Santos et al., 2019), and the impact of individuals’ characteristics on attitudes toward sponsorship-linked marketing (Baek and Reid, 2013). Few studies examine how sponsorship-linked marketing can be fully utilized to increase corporate interest from the perspective of time. This present research discovered that past sponsorship-linked marketing campaigns still maintained value in their ability to attract consumers’ attention. Thus, the conclusions provide a research basis for future studies to explore how past campaigns could sustainably generate revenue and benefits for corporates.

### Practical Implications

First, firms can fully utilize sponsored events information to highlight brand characteristics and value. From the research findings, when presented with past sponsorship-linked marketing campaigns, information on sponsoring brands was conducive to facilitating more positive evaluations of the campaigns. However, information on the sponsored event had no significant impact. As sponsoring firms usually invest huge resources into obtaining naming rights, even after the close of a sponsored event, they could continue to extend their influence by association to further promote brand value. Nevertheless, whether information about the background of the sponsorship campaigns should be introduced requires further consideration. Prior studies show that excessive background information negatively affects consumer evaluations of past events (Zhao et al., 2014). However, it remains prudent to refer to past sponsored events in advertisements and product brochures to emphasize brand value and increase consumers’ positive awareness of the sponsorships.

Second, firms can make rational applications of upcoming sponsorship-linked marketing campaigns and integrate their resources and sponsored events. According to the research findings, information on sponsoring brands and sponsored events for future campaigns, largely influence individuals’ evaluations of events. Therefore, for upcoming campaigns, sponsoring firms should highlight the value and image of the brand and the status, as well as importance of the sponsored event. Moreover, the fit between the brand and the event is particularly important during this process.

### Limitations and Prospects for Future Research

#### Limitations

First, regarding the empirical analysis, the scenarios used in the three studies were invented campaigns adapted from real-life marketing scenarios. Future studies should conduct field research to explore consumers’ true responses under different time-frame stimuli in a real-life context.

Second, although this study considered temporal framing across a range of temporal distances, the different time points were manipulated in the experimental materials. Thus, future research could investigate and compare the effectiveness of other manipulation methods, such as posters of the event and product brochures, to test the hypotheses of this study.

Third, this study referred only to the processing style activation method proposed by McCrea et al. (2012) and did not consider and compare the effectiveness of other activation methods. Future research could apply other methods, such as the Navon task (Navon, 1977), to test the robustness of the findings of this study.

#### Prospects for Future Research

On one hand, according to the findings, consumer preferences for the type of sponsorship information at different time frames varied. However, whether the conclusions can be applied to other contexts requires further examination. For example, future research could investigate the temporal framing effect on individuals’ information preferences in the case of co-branding. Different from sponsorship-linked marketing, none of the brands involved in co-branding serve as a contextual reference for the other. Moreover, co-branding also includes information on the brand status. Hence, it may be valuable to investigate how temporal framing affects individuals’ evaluations in the context of co-branding.

On the other hand, this study only examined the impact of temporal framing on profitable sponsorship-linked marketing campaigns. It may be worth exploring whether the same conclusions can be generalized to non-profit, charitable, or donation events.

## Data Availability Statement

The raw data supporting the conclusion of this article will be made available by the authors, without undue reservation.

## Ethics Statement

Ethical review and approval was not required for the study on human participants in accordance with the local legislation and institutional requirements. This research conducted online-based and paper-pencil tests and was exempt from further ethics board approval since this study did not involve human clinical trials or animal experiments. In the research process, all participants were informed that participation was voluntary and assured that their responses would be used only for academic research and kept strictly confidential. Therefore, only those who were willing to participate were recruited. Participants could withdraw from the study at any time without penalization. All the responses were anonymous, which helped to protect the privacy of participants. Written informed consent from the participants was not required to participate in this study in accordance with the national legislation and the institutional requirements.

## Author Contributions

YZ developed the theory, designed the studies, collected the data, performed data analysis and interpretation, and wrote the manuscript.

## Conflict of Interest

The author declares that the research was conducted in the absence of any commercial or financial relationships that could be construed as a potential conflict of interest.

## Publisher’s Note

All claims expressed in this article are solely those of the authors and do not necessarily represent those of their affiliated organizations, or those of the publisher, the editors and the reviewers. Any product that may be evaluated in this article, or claim that may be made by its manufacturer, is not guaranteed or endorsed by the publisher.
